# Significance of Onodera’s prognostic nutritional index in patients with colorectal cancer: a large cohort study in a single Chinese institution

**DOI:** 10.1007/s13277-015-4008-8

**Published:** 2015-10-05

**Authors:** Chen Jian-hui, Edward Arthur Iskandar, Sh-irong Cai, Chuang-qi Chen, Hui Wu, Jian-bo Xu, Yu-long He

**Affiliations:** 1 Division of Gastrointestinal Surgery Center, The First Affiliated Hospital of Sun Yat-sen University, Guangzhou, 510080 China; 2 Gastric Cancer Center, Sun Yat-sen University, Guangzhou, 510080 China

**Keywords:** Prognostic nutritional index, Clinicopathological feature, Survival, Colorectal cancer

## Abstract

The preoperative nutritional and immunological statuses have an important impact in predicting the survival outcome of patients with various types of malignant tumors. Our study aimed to explore the clinical significance and predictive prognostic potential of Onodera’s prognostic nutritional index (PNI) in patients with colorectal carcinoma. This retrospective study included a total of 1321 patients who were diagnosed with colorectal cancer and who had been surgically treated between January 1994 and December 2007. The PNI level was determined according the following formula: 10 × serum albumin (g/dL) + 0.005 × total lymphocyte count (per mm^3^). The impact of PNI on clinicopathological features and overall survival (OS) was determined. The optimal cutoff value of PNI was set at 45. Patients in the low-PNI group had a greater potential to have aggressive histological features, advanced tumors (T), nodal involvement (N), metastasis (M), and TNM stage than those in the high-PNI group. The low-PNI group had a worse OS than the high-PNI group (5-year survival rate 56.1 vs 64.8 %, respectively; *P* < 0.05). Furthermore, the PNI value was an independent prognostic factor for colorectal cancer in this study. The OS was significantly lower in the low-PNI group than in the high-PNI group in patients with TNM stage II and III diseases. Preoperative PNI is a simple and useful marker to predict clinicopathological features and long-term survival outcome in patients with colorectal carcinoma. PNI analysis should be included in the routine assessment of patients with locally advanced colorectal cancer.

## Introduction

According to GLOBOCAN 2012 database, there were 253,000 new cases of colorectal cancer and 139,000 cases of death due to colorectal cancer; these accounted for 18.6 and 20.0 % of cancers and cancer deaths worldwide, respectively. Moreover, the incidence of new cases of colorectal cancer in China has increased in recent years [1–4]. Surgical resection is the most important method of treatment for colorectal cancer. Hence, it is important to determine preoperative predictive prognostic markers for patients with colorectal cancer who undergo surgical treatment.

The immunological and nutritional statuses have both been deemed as useful preoperative indexes to assess surgical risk, postoperative morbidity, and mortality [5]. An increasing number of studies have shown that immunonutritional status can also be a powerful means of predicting survival outcome [6, 7]. Onodera’s prognostic nutritional index (PNI) is considered a simple and useful index to reflect the preoperative nutritional and immunological condition, and it can be easily calculated using the serum albumin level and peripheral blood total lymphocyte counts. The PNI was originally used to evaluate the risk of postoperative complications and mortality [8] in gastrointestinal tract surgery, and it has become a powerful prognostic parameter for various types of cancer, such as colorectal cancer [9], gastric cancer [10], pancreatic cancer [11], and hepatocellular carcinoma [12]. However, few studies have evaluated the clinical importance of PNI in patients with colorectal cancer in China, and most of the related studies have had a small sample size. Hence, we performed a retrospective analysis to study the clinical importance and prognostic effect of PNI in a large cohort of Chinese patients with colorectal cancer.

## Materials and methods

### Patients

Between January 1994 and December 2007, a total of 1732 patients diagnosed with primary colorectal cancer underwent surgical treatment in the gastrointestinal-pancreatic surgery department of the First Affiliated Hospital of Sun Yat-sen University. Thirty-eight patients with multiple cancer, 249 with incomplete follow-up data, 57 with recurrent colorectal cancer, 155 with incomplete serum albumin data, and 57 with incomplete peripheral blood lymphocyte count data were excluded from this study. At last, 1321 patients were included in this study. Informed consent was obtained from all individual participants included in the study, and this study was approved by the Ethics Committees of the First Affiliated Hospital of Sun Yat-sen University.

### PNI and data

The preoperative peripheral blood samples were collected to obtain the serum albumin and total lymphocyte count. The Onodera’s PNI value was calculated using the following formula: 10 × serum albumin (g/dL) + 0.005 × total lymphocyte count (per mm^3^).

The clinicopathological factors including demographics (age and sex), perioperative blood transfusion, clinical features (tumor size, location, and gross type), histological type, surgical approach, and cancer stage were collected using a larger clinical database of the gastrointestinal-pancreatic surgery department of the First Affiliated Hospital of Sun Yat-sen University since January 1994. Tumor size was determined according to the largest diameter, and patients were divided into two groups based on the median tumor diameter (>5 and <5 cm). We deemed well-differentiated and moderately differentiated adenocarcinoma to be the well-differentiated histological type, and poorly differentiated, undifferentiated, mucinous, and signet ring cell adenocarcinoma to be the poorly differentiated histological type. Tumor stage was classified according to the seventh edition of the American Joint Committee on Cancer (AJCC) TNM classification system.

### Follow-up

The patients received follow-up until December 2014. The patients were followed up every 3 months during the first two postoperative years, every 6 months for the next 3 years, and annually thereafter. Physical examination, chest radiography, colonoscopy, peripheral blood tumor marker measurements (CEA, CA199, CA125, and AFP), and abdominal computed tomography or ultrasonography were performed during the follow-up period. The follow-up rate of this study was 87.7 %.

### Statistical analysis

The statistical analysis was performed using SPSS analysis software program (SPSS 18.0, IBM, Chicago, IL, USA). A receiver operating characteristics (ROC) curve was generated to determine the sensitivity and specificity of the PNI value for predicting 5-year overall survival (OS), and the Youden index was determined to calculate the optimal cutoff value of PNI. The categorical variables are presented as numbers and percentages, and they were compared using the chi-squared or Fisher’s tests. Survival comparison and survival curves were performed and generated using the Kaplan-Meier method, and the 5-, 10-, and 15-year OS rates were calculated using the life-table method. Multivariate and univariate analysis data were obtained from the Cox proportional hazard model. A *P* value less than 0.05 was considered statistically significant.

## Results

### Patient characteristics

A total of 1321 patients with colorectal cancer were analyzed in this study. The baseline clinical and pathological characteristics of all included patients are shown in Table 1. The median age of patients was 57.5 years (range, 18–91 years). There were 777 (58.8 %) men and 544 (41.2 %) women; 609 (46.1 %) patients had colon cancer and 712 (53.9 %) patients had rectal cancer.

**Table 1:** The correlation between PNI status and various of clinicopathological factors

Variable	Low-PNI group	High-PNI group	*χ* ^2^ value	*P* value
Age (years)			10.936^a^	0.001
≤60	253 (46.1 %)	427 (55.3 %)		
>60	296 (53.9 %)	345 (44.7 %)		
Gender			0.646^a^	0.422
Male	330 (60.1 %)	447 (57.9 %)		
Female	219 (39.9 %)	325 (42.1 %)		
Blood transfusion			4.432^a^	0.035
No	412 (75.0 %)	617 (79.9 %)		
Yes	137 (25.0 %)	155 (20.1 %)		
Tumor location			0.272	0.602
Colon	290 (52.8 %)	419 (54.3 %)		
Rectum	259 (47.2 %)	353 (45.7 %)		
Tumor gross types			20.139^a^	<0.001
Mass	226 (41.2 %)	276 (35.8 %)		
Ulcerated	215 (39.2 %)	393 (50.9 %)		
Infiltrative	108 (19.7 %)	103 (13.3 %)		
Histology			5.559^a^	0.018
Well differentiated	429 (78.1 %)	643 (83.3 %)		
Poor differentiated	120 (21.9 %)	129 (16.7 %)		
Tumor size(cm)			26.017^a^	<0.001
≤5	320 (58.3 %)	554 (71.8 %)		
>5	229 (41.7 %)	218 (28.2 %)		
T stage			33.910^a^	0.001
T_1_	20 (3.6 %)	41 (5.3 %)		
T_2_	77 (14.0 %)	170 (22.0 %)		
T_3_	289 (52.6 %)	425 (55.1 %)		
T_4_	163 (29.7 %)	136 (17.6 %)		
N stage			7.066^a^	0.029
N_0_	176 (32.1 %)	302 (39.1 %)		
N_1_	173 (31.5 %)	224 (29.0 %)		
N_2_	200 (36.4 %)	246 (31.9 %)		
M stage			9.796^a^	0.002
M_0_	420 (76.5 %)	644 (83.4 %)		
M_1_	129 (23.5 %)	128 (16.6 %)		
TNM stage			18.736^a^	0.001
I	52 (9.5 %)	125 (16.2 %)		
II	205 (37.3 %)	286 (37.0 %)		
III	163 (29.7 %)	233 (30.2 %)		
IV	129 (23.5 %)	128 (16.6 %)		
Dukes stage			13.762^a^	0.003
A	46 (8.4 %)	95 (12.3 %)		
B	130 (23.7 %)	207 (26.8 %)		
C	244 (44.4 %)	342 (44.3 %)		
D	129 (23.5 %)	128 (16.6 %)		

### ROC curve analysis

**Fig. 1:**
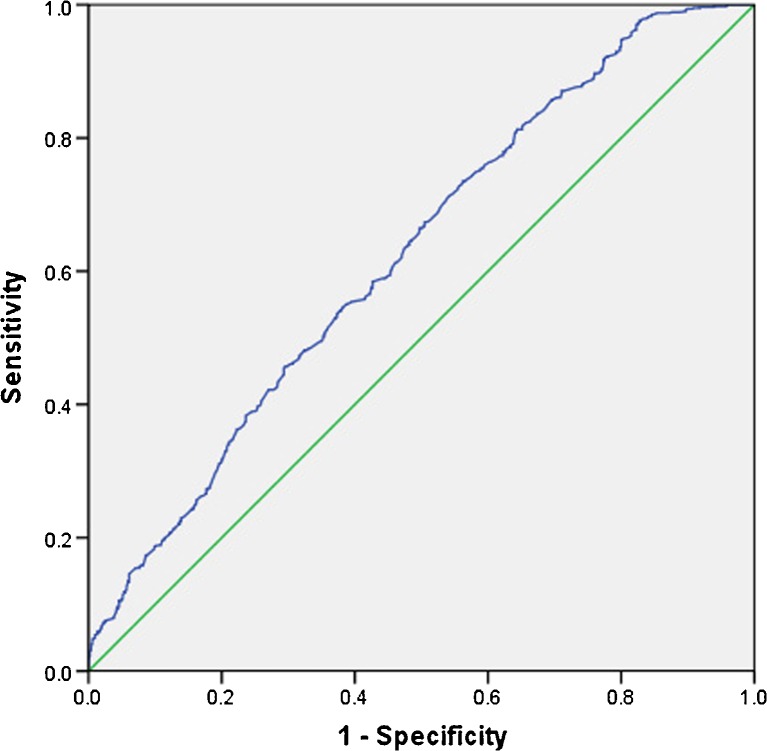
Receiver operating characteristic (ROC) curve analysis for the prognostic nutritional index. When the PNI value was 45, the Youden index was maximal (0.169) and the sensitivity and specificity for 5-year OS were 0.849 and 0.690, respectively

The mean value of PNI was 50.2 (range, 24.9–70.0). According to 5-year survival rate, the area under the ROC curve for the PIN was 0.625 (*P* < 0.001). When the PNI value was 45, the Youden index was maximal and the sensitivity and specificity for 5-year OS were 0.849 and 0.690, respectively (Fig. 1). Hence, we divided the patients into two groups based on the previously described optimal cutoff value of PNI: the high-PNI group (PNI ≥ 45, *n* = 772) and the low-PNI group (PNI < 45, *n* = 549).

### Association between PNI and clinicopathological features

The association between PNI status and various clinicopathological factors is shown in Table 1. Among all 1321 patients, there were no significant distribution differences in sex and tumor location between the two groups. Older age (age ≥60 years), larger tumor size, worse gross type, and poorly differentiated histological type were more frequently observed in the low-PNI group. Additionally, patients in the low-PNI group had a higher incidence of perioperative blood transfusion. Moreover, patients in the low-PNI group were more likely to have advanced tumors (T), nodal involvement (N), metastasis (M), TNM stage, and Dukes stage.

### Association between PNI and survival

During the follow-up period, the median survival time of all patients was 86.3 months and the average survival time was 88.6 months (range, 1.0–235.0 months). At the last follow-up, 766 (58.0 %) patients had died. The 5-, 10-, and 15-year OS rates were 61.2, 45.6, and 35.9, respectively.

**Fig. 2:**
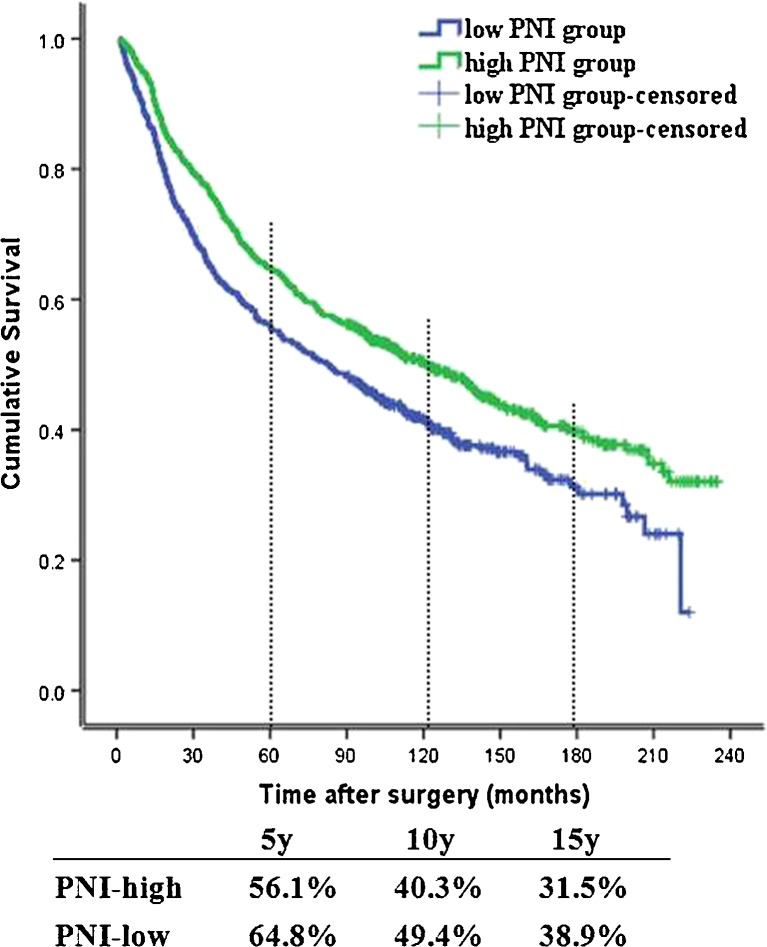
Kaplan-Meier analysis of overall survival (OS) for a total of 1321 cases with colorectal cancer according to the prognostic nutritional index (PNI) value. The low-PNI group had a worse OS rate than the high-PNI group (*P* < 0.05)

The relationship between PNI and OS is presented in Fig. 2. A low PNI value was associated with reduced OS (*P* < 0.05). The 5-, 10-, and 15-year OS rates were 56.1, 40.3, and 31.5 % in the low-PNI group and 64.8, 49.4, and 38.9 % in the high-PNI group, respectively.

**Fig. 3:**
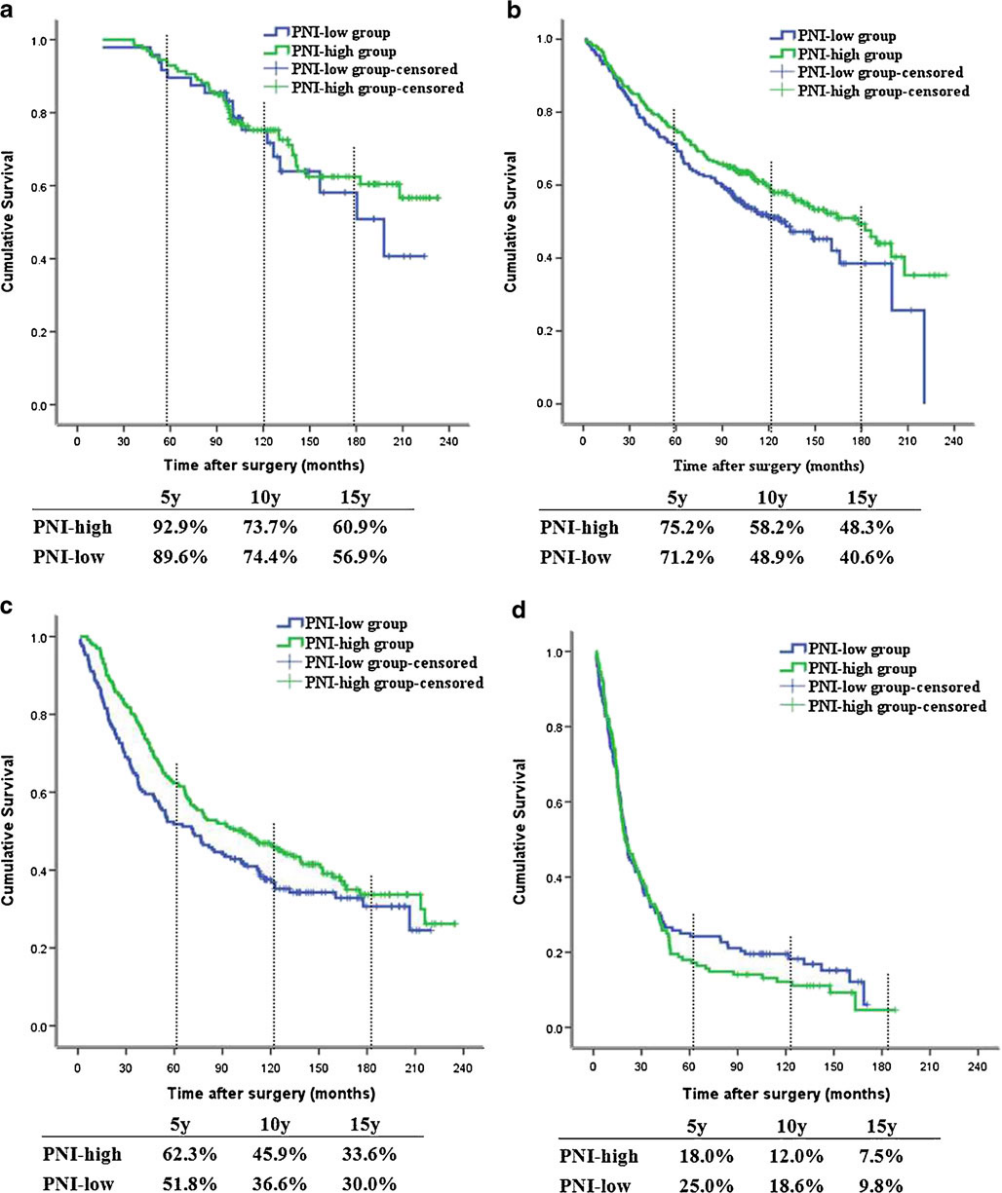
Kaplan-Meier analyses of overall survival (OS) for a total of 1321 patients with colorectal cancer according to the prognostic nutritional index (PNI) value among patients with stage I (**a**, *P* = 0.535), stage II (**b**, *P* = 0.042), stage III (**c**, *P* = 0.049), and stage IV (**d**, *P* = 0.454) disease. Patients with stage II and III disease in the high-PNI group had a better survival outcome than those in the low-PNI group

To further investigate the consistency of PNI in different TNM stages, we found no survival differences among patients with TNM I and IV stages between the two groups (*P* = 0.535 and 0.454, respectively, shown in Fig. 3a, d). Furthermore, patients in the high-PNI group had a better OS than those in the low-PNI group with TNM II and III stages (*P* = 0.042 and 0.049, respectively, shown in Fig. 3b, c).

### Prognostic significance of PNI for overall survival

Results of univariate analysis for OS are shown in Table 2, which shows the factors associated with a statistically worse OS, such as age ≥60 years, perioperative blood transfusion, larger tumor size ≥5 cm, infiltrative gross type, poor histological type, advanced T stage [T4 vs (T3 + T2 + T1)], lymph node involvement, distant metastasis, advanced TNM stage [(III + IV) vs (I + II)], advanced Dukes stage [(C + D) vs (A + B)], palliative surgery, and low PNI value; sex and tumor location were not associated with survival. Multivariate analysis revealed that only the low PNI value (hazard ratio, 0.862; 95 % confidence interval, 0.527–0.932; *P* = 0.014), infiltrative gross type, advanced T stage, lymph node involvement, and distant metastasis were independent prognostic factors for adverse OS of colorectal cancer patients in this study.

**Table 2:** Univariate and multivariate analyses for overall survival of 1321 cases with colorectal cancer

Factor	Univariate analyses			Multivariate analyses		
	*χ* ^2^ value	HR	*P* value	*χ* ^2^ value	HR	*P* value
Age (>60 years)	21.889	1.405	0.001			
Gender (male)	0.023		0.878			
Blood transfusion (yes)	25.859	1.453	0.001			
Tumor location (colon)	2.441		0.118			
Size (>5 cm)	4.236	1.169	0.04			
Gross type (ulcerated)	24.112	1.279	0.001	11.069	1.191	0.001
Histological type (poor differentiated)	23.434	1.521	0.001			
T stage (T_3_ + T_4_)	62.262	1.472	0.001	7.111	1.147	0.008
N stage (N_1~3_)	93.467	1.393	0.001	6.494	1.107	0.011
M stage (M_1_)	224.747	3.374	0.001	43.942	2.032	0.001
TNM stage (III + IV stage)	198.582	1.789	0.001			
Dukes stage (C + D stage)	238.757	1.903	0.001			
Surgical approach (radical)	241.337	5.463	0.001	102.463	3.367	0.001
PNI (<45)	14.258	0.759	0.001	3.953	0.862	0.047

*HR* hazard ratio

## Discussion

The PNI, a simple and useful systemic inflammation-based prognostic score, is calculated based on laboratory assessments of total lymphocyte count and serum albumin level [8] and can reflect the pretreated host immunological and nutritional status. Low PNI was first found to be a predictor of a high risk of short-term postoperative complications in the gastrointestinal tract. Recently, increasing evidence revealed that low PNI was also related to reduced survival in various types of malignant tumors [10–12]. In this retrospective study, we demonstrated that low PNI was associated with older ages and aggressive clinicopathological features, which led to a worse overall survival for patients with colorectal cancer. Hence, from the multivariate analysis, PNI was a worse independent prognostic factor in our study.

It was widely accepted that the host-related inflammatory response plays an important role in tumorigenesis, tumor progression, and metastasis through recruitment of regulatory T lymphocytes and cytokines, including interleukin-1 (IL-1), IL-6, and tumor necrosis factor α [13]. Moreover, in patients with malignant tumors, the presence of inflammation can be reflected by various preoperative hematological inflammation-based prognostic scores [14], such as the neutrophil-to-lymphocyte ratio, platelet-to-lymphocyte ratio, Glasgow Prognostic Score, and PNI. However, few studies have investigated the clinical impact of PNI on the clinicopathological features and survival outcome of patients with colorectal cancer.

In previous reports, various cutoff values have been used for PNI. Some Japanese authors set the PNI cutoff value at 40 for patients with colorectal cancer [15–17]. Ikeya found the optimal cutoff value for PNI was 44.5 according to the ROC curve analysis [18]. However, for other kinds of malignant tumors, most studies usually set the cutoff value of PNI at 45, because PNI < 45 was regarded as malnutrition and was accompanied by a high risk of postoperative complications. In our large cohort study, with the help of the ROC curve analysis for the 5-year overall survival of colorectal cancer patients, we found that the optimal cutoff point for PNI was 45, similar to the result of Mohri’s study [9].

Nozoe et al. retrospectively analyzed 219 patients with colorectal cancer [15]. Their study revealed no association between PNI and lymph node involvement, and a close relationship between PNI and TNM stage that may be reflected by the tumor depth. Our study also demonstrated the significant value of PNI in predicting the aggressive clinicopathological features of patients with colorectal cancer, including advanced tumor depth, lymph node involvement, distant metastasis, and TNM classification.

The initial aim of the Onodera PNI was to evaluate the nutritional and immunological status to predict the short-term postoperative outcome of patients with gastrointestinal malignancy. Increasing evidence reported that PNI had a prognostic impact on the long-term survival outcome of patients in with numerous malignant carcinomas. Hong found that the low PNI group had a worse median OS time and that low PNI was an independent significant prognostic factor for worse OS [19]. Similarly, Ishizuka revealed that patients with gastric cancer and higher PNI (>45) had a better postoperative survival rate after total gastrectomy [20]. Our multivariate Cox analysis clearly demonstrated that PNI was an independent prognostic survival factor for patients with colorectal cancer. Although some research has confirmed the clinical impact of PNI in patients with unresectable metastatic colorectal cancer, in our study, no survival difference was observed in the OS rate with respect to the different pretreated PNI levels for those with stage IV disease. Furthermore, we found that the OS was significantly worse in the low-PNI group only among those with stages II and III disease. A similar result was found by Jiang et al. [21], who found that when the cutoff value of PNI was set at 46, the 5-year survival rates were significantly lower in the low-PNI group among patients with stage II and III disease. These results suggested that low PNI might be associated with a worse prognosis in patients with locally advanced cancer.

Some limitations to this study should be acknowledged. First, this was a retrospective analysis, and some preoperative hematological data were lost. Nevertheless, the large sample size provided adequate statistical efficiency to explore the impact of the PNI value on clinicopathological features and long-term OS. Moreover, this study excluded patients who received neoadjuvant chemotherapy or chemoradiotherapy, which may have led to a negative impact on the survival outcome. Finally, this was not a continuous study because there were a number of cases with missing data due to incomplete serum albumin or peripheral blood lymphocyte count data. Hence, further research is needed to confirm these preliminary findings.

From this retrospective study, we can conclude that PNI is a simple and effective marker for predicting the aggressive clinicopathological features and long-term survival outcome in patients with colorectal cancer.
